# A phase I/randomized phase II study of GM.CD40L vaccine in combination with CCL21 in patients with advanced lung adenocarcinoma

**DOI:** 10.1007/s00262-018-2236-7

**Published:** 2018-09-12

**Authors:** Jhanelle E. Gray, Alberto Chiappori, Charlie C. Williams, Tawee Tanvetyanon, Eric B. Haura, Ben C. Creelan, Jongphil Kim, Theresa A. Boyle, Mary Pinder-Schenck, Farah Khalil, Soner Altiok, Rebecca Devane, David Noyes, Melanie Mediavilla-Varela, Renee Smilee, Emily L. Hopewell, Linda Kelley, Scott J. Antonia

**Affiliations:** 1 Department of Thoracic Oncology, H. Lee Moffitt Cancer Center and Research Institute, 12902 Magnolia Drive, FOB1, Tampa, FL 33612 USA; 2 Department of Biostatistics and Bioinformatics, H. Lee Moffitt Cancer Center and Research Institute, Tampa, FL USA; 3 Department of Anatomic Pathology, H. Lee Moffitt Cancer Center and Research Institute, Tampa, FL USA; 4 GlaxoSmithKline, Collegeville, PA USA; 5 Clinical Trials Office, H. Lee Moffitt Cancer Center and Research Institute, Tampa, FL USA; 6 Clinical Science Lab (Antonia Lab), H. Lee Moffitt Cancer Center and Research Institute, Tampa, FL USA; 7 Cell Therapy Core, H. Lee Moffitt Cancer Center and Research Institute, Tampa, FL USA

**Keywords:** Cancer vaccine, Chemokine, Immunotherapy, Non-small cell lung cancer

## Abstract

The GM.CD40L vaccine, which recruits and activates dendritic cells, migrates to lymph nodes, activating T cells and leading to systemic tumor cell killing. When combined with the CCL21 chemokine, which recruits T cells and enhances T-cell responses, additive effects have been demonstrated in non-small cell lung cancer mouse models. Here, we compared GM.CD40L versus GM.CD40L plus CCL21 (GM.CD40L.CCL21) in lung adenocarcinoma patients with ≥ 1 line of treatment. In this phase I/II randomized trial (NCT01433172), patients received intradermal vaccines every 14 days (3 doses) and then monthly (3 doses). A two-stage minimax design was used. During phase I, no dose-limiting toxicities were shown in three patients who received GM.CD40L.CCL21. During phase II, of evaluable patients, 5/33 patients (15.2%) randomized for GM.DCD40L (*p* = .023) and 3/32 patients (9.4%) randomized for GM.DCD40L.CCL21 (*p* = .20) showed 6-month progression-free survival. Median overall survival was 9.3 versus 9.5 months with GM.DCD40L versus GM.DCD40L.CCL21 (95% CI 0.70–2.25; *p* = .44). For GM.CD40L versus GM.CD40L.CCL21, the most common treatment-related adverse events (TRAEs) were grade 1/2 injection site reaction (51.4% versus 61.1%) and grade 1/2 fatigue (35.1% versus 47.2%). Grade 1 immune-mediated TRAEs were isolated to skin. No patients showed evidence of pseudo-progression or immune-related TRAEs of grade 1 or greater of pneumonitis, endocrinopathy, or colitis, and none discontinued treatment due to toxicity. Although we found no significant associations between vaccine immunogenicity and outcomes, in limited biopsies, one patient treated with GMCD40L.CCL21 displayed abundant tumor-infiltrating lymphocytes. This possible effectiveness warrants further investigation of GM.CD40L in combination approaches.

**Electronic supplementary material:**

The online version of this article (10.1007/s00262-018-2236-7) contains supplementary material, which is available to authorized users.

## Introduction

Novel approaches are needed for patients with advanced lung adenocarcinoma, who continue to have poor outcomes despite refinements in chemotherapeutic regimens. For patients with advanced non-small cell lung cancer (NSCLC), immunotherapeutic approaches are available due to the success of the US Food and Drug Administration (FDA)-approved PD-1/PD-L1 inhibitors pembrolizumab [1], nivolumab [2–5], and atezolizumab [6, 7]. Another potential immunotherapeutic approach is cancer vaccines, which can stimulate the immune system by expanding tumor-reactive T-cell numbers to achieve improved patient outcomes.

GM.CD40L, a human bystander cell line created at the Moffitt Cancer Center that expresses both granulocyte-macrophage colony-stimulating factor (GM-CSF) and CD40 ligand (CD40L), has been used to generate an allogeneic tumor cell-based vaccine formulation. The bystander cells help to recruit professional antigen-presenting cells in the form of dendritic cells (DCs) by secreting GM-CSF in the vaccine site microenvironment. Once activated by CD40L, DCs take up apoptotic bodies from the irradiated tumor cells in the vaccine and present tumor antigens in the context of the major histocompatibility complex proteins. These activated DCs, now loaded with tumor antigens, migrate to regional lymph nodes, where T-cell activation occurs. Activated tumor antigen-specific T cells can then exit the lymph node and recirculate to the tumor, leading to systemic tumor cell killing.

**Fig. 1:**
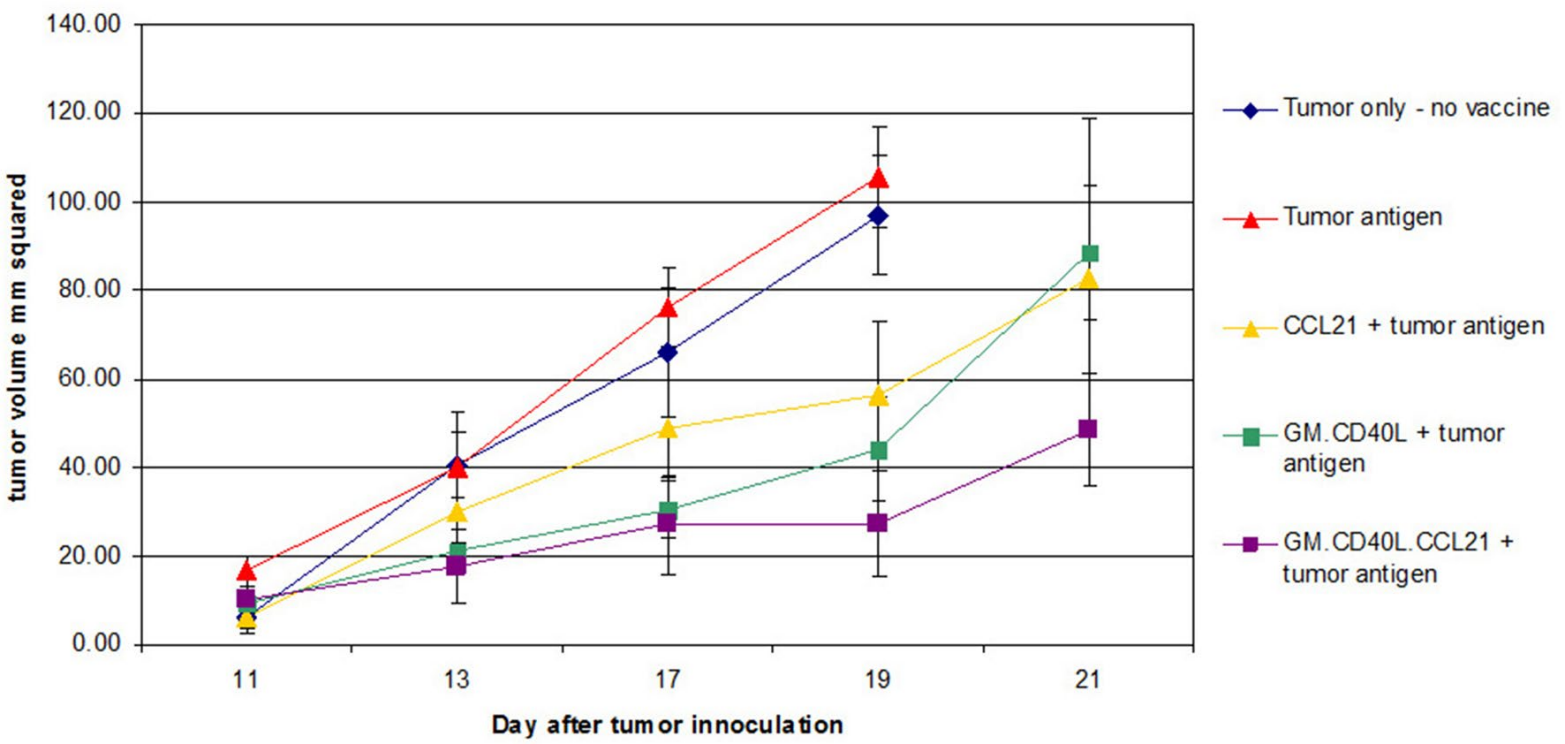
Treatment of Lewis lung cancer mouse models with GM.CD40L plus CCL21 decreases tumor volumes and increases time to progression. Mice were inoculated with tumor cells on day 0 and vaccinated on day 5 and then three more times every 3 to 4 days. Tumor volume was measured. At the end of the study, lymph-node cells and splenocytes were harvested. Time to tumor progression increased significantly in all vaccine-treated mice; however, mice treated with GM.CD40L.CCL21 had a longer time to progression and an overall smaller tumor volume (*p* = .038)

In trials of the vaccine plus cyclophosphamide or all-trans-retinoic acid (ATRA) for reduction of T-regulatory cells [8] or induction of myeloid-derived suppressor cell differentiation [9, 10], patients with advanced NSCLC showed a median progression-free survival (PFS) of 1.7 months and median overall survival (OS) of 7.9 months. Although outcomes were similar to those of patients treated with pemetrexed and docetaxel [11], adverse effects, such as headaches (54%), due to addition of the cyclophosphamide and ATRA, were agreed to be too burdensome on patients. CCL21, a chemokine that helps recruit T cells and leads to hyper-responsive T cells, may help to enhance the GM.CD40L vaccine. In preclinical studies, CCL21 combined with costimulatory molecules showed synergistic anti-tumor effects [12], increasing interferon-γ-producing CD8+ cells while inducing apoptosis in CD4+CD25+FoxP3+ regulatory T cells [13]. In unpublished experiments, we observed improved time to progression in Lewis lung cancer mouse models given GM.CD40L vaccine plus CCL21 versus vaccine alone (Fig. 1).

On the bases of improved outcomes in the animal model shown with GM.CD40L plus CCL21 and the limited options for previously treated patients with advanced or stage IV lung adenocarcinoma, we conducted a single-center phase I/randomized phase II trial to evaluate GM.CD40L vaccine plus/minus CCL21.

## Materials and methods

### Patients and sites

This single-institution study (NCT01433172) enrolled patients with advanced/metastatic lung adenocarcinoma who had no curative options. Inclusion criteria included receipt of at least one prior line of therapy, an Eastern Cooperative Oncology Group performance status of 0 to 1, no significant laboratory abnormalities, life expectancy of > 6 months, and measurable disease by Response Evaluation Criteria In Solid Tumors (RECIST) version 1.1. Patients with history of chronic steroid therapy (prednisone > 10 mg), who had pre-existing autoimmune disorders or who were pregnant or breastfeeding, were excluded. Prior immunotherapy was allowed.

### Study design

In the phase I portion, three patients were treated with GM.CD40L plus CCL21 (GM.CD40L.CCL21). The dose-limiting toxicity (DLT) period was 28 days. If a single patient experienced grade 3 hematologic or non-hematologic toxicities or any grade 2 immune-related toxicity (except fever), three additional patients would be added at the same dose. The dose of the vaccine was not escalated beyond 3 × 10^7^ cells/injection; therefore, the maximum tolerated dose may not have been reached in this study.

The recommended phase II dose (RP2D) was defined as the highest GM.CD40L.CCL21 vaccine dose level that induced DLT in fewer than 33% of patients. Once the GM.CD40L.CCL21 RP2D was established, the phase II portion was initiated, with patients randomized 1:1 to receive GM.CD40L or GM.CD40L.CCL21. Patients were stratified by age and sex. Data on subsequent therapies were collected.

### Treatments and efficacy assessments

Patients who received GM.CD40L alone received three vaccinations, which included irradiated H1944 adenocarcinoma cells, irradiated H2122 adenocarcinoma cells, and GM.CD40L cells. Patients in the combination group received GM.CD40L plus H1944 cells, which was different from the other vaccine in that the H1944 cells now expressed CCL21. Patients were injected intradermally in four separate sites (0.25 mL/site), at bilateral proximal upper and lower extremities (axillary and inguinal nodal basins).

Patients in both groups received vaccines every 14 days for three immunizations, then every 28 days for three immunizations, and then booster vaccines every 3 months until disease progression, unacceptable toxicity, or patient withdrawal.

Efficacy assessments (using RECIST version 1.1) occurred at baseline and at days 42 and 133. Patients who received booster vaccines were also assessed on days 196, 273, and 367. Patients were allowed to remain on treatment if there were no signs of clinical progression.

### Toxicity

Adverse events (AEs) were graded according to the National Cancer Institute (NCI) Common Toxicity Criteria for Adverse Events version 4. Serious adverse events were also collected for analysis.

### Vaccine production and release criteria

The GM.CD40L bystander cell line [14] and the NCI-H1944 and NCI-H2122 cell lines [10] were prepared as previously described. Vaccines were produced as previously described with minor changes [10]. Ad-CCL21 (a replication-defective adenovirus vector propagated in E1A complementing cell lines) was obtained from NCI-Frederick. The CCL21 transgene resides in the E1A locus of the adenovirus. A 0.1-mL sample of the vaccine was removed for final release testing, with release criteria of “no organisms seen” on Gram stain and inoculation of blood culture bottles for 14-day sterility testing.

### Correlative studies

Several tumor-associated antigens over-expressed in NSCLC lines have been identified, including CEA and WT-1 [15, 16]. When available, pre-treatment archival tissue was collected to determine whether anti-tumor responses correlated with proteins of interest (that is, shared tumor antigens known to be expressed by the allogeneic tumor cells in the vaccine).

Immunohistochemistry was as previously described: anti-CD3 antibody (prediluted, 790-4341, Ventana, Tucson, Arizona) [17], anti-CD45 antibody (#ab10558, 1:200 dilution, Abcam, Cambridge, Massachusetts) [18], anti-WT1 antibody C-19 (Santa Cruz Biotechnology, Santa Cruz, CA) [19], anti-CEA antibody 12.140.10 (Novocastra Labs, United Kingdom) [20], anti-hTERT antibody 44F12 (Novocastra Labs) [21], and anti-PD-L1 antibody AT-0713-000362 (Sino Biological Inc, China) [22]. Scoring for PD-L1 was done by the total proportion score, which is the percentage of tumor cells with any intensity of positive membranous staining (score ≥ 50%, defined as positive in accordance with the guidelines established by Merck). The exploratory biomarkers WT1, CEA, and hTERT were assessed with histology score (H-score), a semi-quantitative scoring system for protein expression. The H-score calculation is based on tumor cell staining percentage and tumor cell staining intensity and calculated as follows: H-score = (percentage faintly stained) + 2 × (percentage moderately stained) + 3 × (percentage strongly stained). The H-score ranges from 0 (no expression in any tumor cells) to 300 (strong expression in 100% of tumor cells). There are few publications and no established guidelines for positivity of WT1, CEA, and hTERT; therefore, we used an H-score cutoff of ≥ 0 for WT1, because most results were 0, and used median scores for CEA (≥ 125) and for hTERT (≥ 100) to designate tumors as “negative” or “positive.” Vaccine immunogenicity was measured by in vitro testing of serial peripheral blood lymphocytes for cytokine-secreting T cells in ELISPOT (enzyme-linked immunospot) assays. Because responses may depend on HLA typing, HLA-A0201 was determined at baseline by flow cytometry followed by molecular analysis of a peripheral blood specimen; however, this result was not an inclusion criterion.

### Statistical analyses

Using a standard 3 + 3 design, the primary phase I endpoint was safety and tolerability. The primary phase II endpoint was 6-month PFS, as this provided a boundary for the go/no-go decision. Assumptions were based on historical data, with rates of ≤ 5% for 6-month PFS designated as not warranting further study and 20% for 6-month PFS designated  as a promising result to pursue further study. For each cohort, we used the two-stage Simon Minimax design [23] with 10% type I and 10% type II error rates to determine sample size, with 18 patients enrolled in the first stage of the trial with 10% rejection error. If 1 or more patients were progression-free at 6 months, 14 additional patients (a total of 32 patients per group) were to be enrolled. If the total number of patients who were progression-free was greater than or equal to 4, the null hypothesis was to be rejected.

All patients who underwent randomization were considered in the PFS (time from start of treatment to progression or death) and OS (time from initiation of treatment to death) analyses. Toxicity assessments included all patients who received at least one vaccine. Follow-up for these analyses continued for all patients for their lifetimes. The final phase II analysis was conducted after follow-up of at least 6 months for all patients who were progression-free; 1 patient was progression free at 6 months; thus, the trial met this endpoint. The primary endpoint was assessed by the Atkinson and Brown [24] method to take into account the nature of two-stage design. The difference between the two treatment groups was assessed by Cochran–Mantel–Haenszel method and two-way analysis of variance to adjust for the effect of stratification variables (sex and age < 70 or ≥ 70 years), respectively. PFS and OS were calculated by the Kaplan–Meier method, with their differences examined by the stratified log-rank test. The hazard ratio between two groups was estimated by the stratified Cox regression model. Correlations between tissue biomarkers and outcomes were computed by Mantel–Haenszel test. All statistical analyses were conducted using SAS statistical software version 9.4 (Cary, North Carolina).

## Results

Between 4/2012 and 1/2016, we enrolled 79 patients, with 73 receiving at least one vaccine dose (Supplemental Fig. 1). In phase I, 3 patients were treated with GM.CD40L.CCL21. The RP2D of the combination vaccine was 7.5 × 10^6^ irradiated H1944 adenocarcinoma cells expressing CCL21 (multiplicity of infection of 500), 7.5 × 10^6^ irradiated H2122 adenocarcinoma cells, and 15 × 10^6^ GM.CD40L cells (1.1 mL). In phase II, 37 and 33 patients were randomized to GM.CD40L (Arm 1) versus GM.CD40L.CCL21 (Arm 2), respectively. For analysis purposes, patients receiving the same GM.CD40L.CCL21 treatment in phase I or phase II were grouped together. The baseline characteristics were balanced between the two groups with no statistically significant differences (Table 1). All patients had received extensive prior therapy (median of three lines of therapy).

**Table 1:** Patient characteristics

	No. of patients (%)		Total	*p* value
	GM.CD40L (*n* = 37)	GM.CD40L.CCL21 (phase I + II; *n* = 36)		
Median age (range), years	69 (38–86)	69 (48–82)		.57
Sex				NA
Female	19 (51.4%)	19 (52.8%)	38	
Male	18 (48.6%)	17 (47.2%)	35	
ECOG performance status				.74
0	13 (35.1%)	12 (33.3%)	25	
1	24 (64.9%)	24 (66.7%)	48	
Race				.59
White	35 (94.6%)	34 (94.4%)	69	
Black	1 (2.7%)	1 (2.8%)	2	
Asian/Pacific Islander	0 (0%)	1 (2.8%)	1	
Unknown	1 (2.7%)	0 (0%)	1	
Ethnicity				.55
Hispanic	1 (2.7%)	2 (5.6%)	3	
Non-Hispanic	36 (97.3%)	34 (94.4%)	70	
Smoking				.58
Current	2 (5.4%)	1 (2.8%)	3	
Former	26 (70.3%)	23 (63.9%)	49	
Never	9 (24.3%)	12 (33.3%)	21	
EGFR mutant	5 (13.5%)	8 (22.2%)	13	.31
KRAS mutant	7 (18.9%)	8 (22.2%)	15	.30
ALK mutant	0 (0%)	1 (2.8%)	1	.64
Median number of prior treatments (range)	3 (1–9)	2.5 (1–6)		.96

### Safety

No DLTs were observed in phase I. The study did not meet the early stopping rule of toxicity. The frequency of AEs of any cause or with any grade was similar between the two groups (81% for GM.CD40L and 92% for GM.CD40L.CCL21; *p* = .19). The most common treatment-related adverse events (TRAEs) for GM.CD40L were grade 1–2 injection site reaction (51.4%), fatigue (35.1%), and anorexia (13.5%) (Table 2). For GM.CD40L.CCL21, the most frequent TRAEs were grade 1 injection site reaction (61.1%) and grade 1–2 fatigue (47.2%) and anorexia (19.4%). Overall, the only immune-mediated TRAEs observed were mild and isolated to the skin: 5.4% versus 5.6% grade 1 pruritus and 5.4% vs. 2.8% dry mouth for GM.CD40L versus GM.CD40L.CCL21. There were no immune-related TRAEs of ≥ grade 1 of pneumonitis, endocrinopathy, hepatitis, neuritis, nephritis, or colitis. No serious adverse events or deaths related to the vaccine were reported, and no patients discontinued treatment due to toxicity.

**Table 2:** Treatment-related adverse events occurring in ≥ 5% of patients

Adverse event	Number (%)			
	Grade 1	Grade 2	Grades 3–5	Total
GM.CD40L (*n* = 37)				
Injection site reaction	18 (48.6)	1 (2.7)	0 (0)	19 (51.4)
Fatigue	8 (21.6)	5 (13.5)	0 (0)	13 (35.1)
Anorexia	4 (10.8)	1 (2.7)	0 (0)	5 (13.5)
Headache	3 (8.1)	0 (0)	0 (0)	3 (8.1)
Hyperkalemia	3 (8.1)	0 (0)	0 (0)	3 (8.1)
Nausea	2 (5.4)	1 (2.7)	0 (0)	3 (8.1)
Edema limbs	3 (8.1)	0 (0)	0 (0)	3 (8.1)
Generalized muscle weakness	2 (5.4)	1 (2.7)	0 (0)	3 (8.1)
Alkaline phosphatase increased	1 (2.7)	1 (2.7)	0 (0)	2 (5.4)
Anemia	1 (2.7)	1 (2.7)	0 (0)	2 (5.4)
Constipation	2 (5.4)	0 (0)	0 (0)	2 (5.4)
Cough	1 (2.7)	1 (2.7)	0 (0)	2 (5.4)
Diarrhea	2 (5.4)	0 (0)	0 (0)	2 (5.4)
Dizziness	2 (5.4)	0 (0)	0 (0)	2 (5.4)
Dry mouth	2 (5.4)	0 (0)	0 (0)	2 (5.4)
Dry skin	2 (5.4)	0 (0)	0 (0)	2 (5.4)
Pruritus	2 (5.4)	0 (0)	0 (0)	2 (5.4)
GM.CD40L.CCL21 (phase I + II; *n* = 36)				
Injection site reaction	22 (61.1)	0 (0)	0 (0)	22 (61.1)
Fatigue	13 (36.1)	4 (11.1)	0 (0)	17 (47.2)
Anorexia	5 (13.9)	2 (5.6)	0 (0)	7 (19.4)
Back pain	4 (11.1)	0 (0)	0 (0)	4 (11.1)
Bone pain	2 (5.6)	2 (5.6)	0 (0)	4 (11.1)
Headache	2 (5.6)	2 (5.6)	0 (0)	4 (11.1)
Aspartate aminotransferase increased	2 (5.6)	1 (2.8)	0 (0)	3 (8.3)
Constipation	2 (5.6)	1 (2.8)	0 (0)	3 (8.3)
Diarrhea	3 (8.3)	0 (0)	0 (0)	3 (8.3)
Dyspnea	1 (2.8)	2 (5.6)	0 (0)	3 (8.3)
Hyperkalemia	3 (8.3)	0 (0)	0 (0)	3 (8.3)
Nausea	2 (5.6)	1 (2.8)	0 (0)	3 (8.3)
Pain in extremity	3 (8.3)	0 (0)	0 (0)	3 (8.3)
Edema limbs	3 (8.3)	0 (0)	0 (0)	3 (8.3)
Alanine aminotransferase increased	2 (5.6)	0 (0)	0 (0)	2 (5.6)
Alkaline phosphatase increased	2 (5.6)	0 (0)	0 (0)	2 (5.6)
Bruising	2 (5.6)	0 (0)	0 (0)	2 (5.6)
Dry skin	2 (5.6)	0 (0)	0 (0)	2 (5.6)
Fever	2 (5.6)	0 (0)	0 (0)	2 (5.6)
Hyponatremia	2 (5.6)	0 (0)	0 (0)	2 (5.6)
Peripheral sensory neuropathy	2 (5.6)	0 (0)	0 (0)	2 (5.6)
Pruritus	2 (5.6)	0 (0)	0 (0)	2 (5.6)

30/37 (81%) patients who received GM.CD40L experienced at least one treatment-related adverse event, whereas 33/36 (92%) patients in the combination vaccine group (GM.CD40L.CCL21) experienced at least one treatment-related AE. The difference between the two groups was not significant (*p* = .19)

### Efficacy

**Fig. 2:**
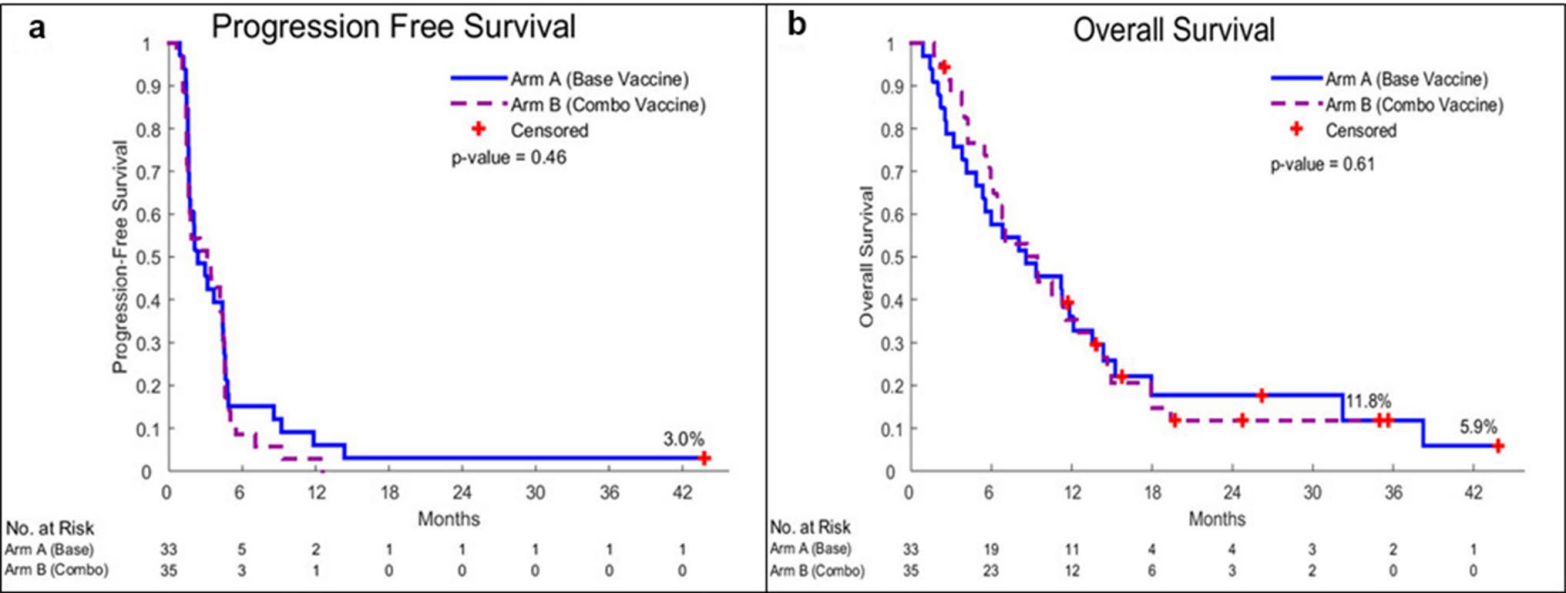
Overall survival and progression-free survival curves. Kaplan–Meier progression-free survival (PFS) curves (**a**) and overall survival (OS) curves (**b**) are presented for the intent-to-treat patient population. Blue is GM.CD40L. Purple is GM.CD40L.CCL21

Four of thirty-seven patients on GM.DCD40L (1 ineligible; 3 withdrew consent) and 1/33 patients on GM.CD40L.CCL21 (lacking documented measurable disease) in the randomized phase II portion were not evaluable for PFS and were thus replaced. For the primary endpoint (6-month PFS), 33 patients who received GM.DCD40L and 32 who received GM.DCD40L.CCL21 were evaluable. Five patients (15.2%) in the GM.DCD40L group (*p* = .023) and three patients (9.4%) in the GM.DCD40L.CCL21 group (*p* = .20) showed 6-month PFS. Median PFS was 2.4 months [95% confidence interval (CI), 1.6–4.4] versus 3.4 months (hazard ratio of 0.87; 95% CI 0.52–1.45; *p* = .61), respectively (Fig. 2a). Median OS was 9.3 versus 9.5 months for patients receiving GM.DCD40L versus GM.DCD40L.CCL21 (hazard ratio of 1.25; 95% CI 0.70–2.25; *p* = .44) (Fig. 2b). When we analyzed patients in the GM.DCD40L.CCL21 arm combined with the 3 patients on the phase I portion, the 6-month PFS, median PFS, and median OS were 8.6%, 3.1, and 9.4 months, respectively. Of 32 patients who received GM.DCD40L versus 35 patients who received GM.DCD40L.CCL21 evaluable by RECIST v1.1, 47% versus 37% had stable disease and 53% versus 63% had progressive disease. No objective responses were observed. Regardless of treatment arm, all nine patients who remained on treatment after progression demonstrated confirmed progression on succeeding scans and discontinued treatment. 11 patients on GM.DCD40L (2 chemotherapy, 4 immunotherapy, 2 investigational agents, and 3 epidermal growth factor receptor-tyrosine kinase inhibitor-based therapy) and 12 patients on GM.DCD40L.CCL21 (5 chemotherapy, 1 investigational agent, 2 immunotherapy, and 4 epidermal growth factor receptor-tyrosine kinase inhibitor-based therapy) received subsequent lines of therapy. In both arms, there were no significant differences in OS between patients who received subsequent immunotherapy and those who did not (*p* = .43 and *p* = .69, respectively).

### Correlative analyses

**Fig. 3:**
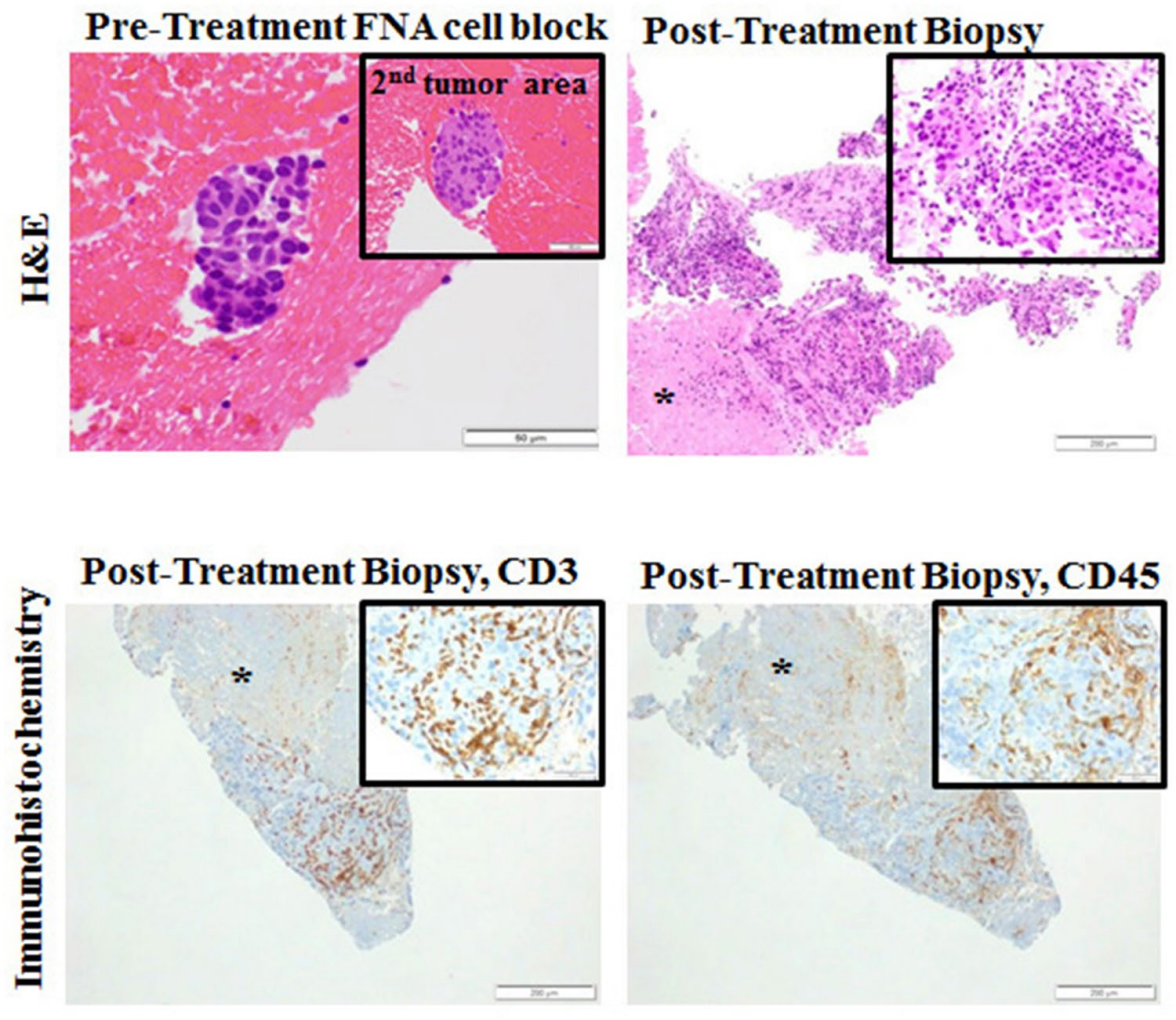
Histopathology results from patient 3 (with progressive disease, treated on phase 1 after 3 induction doses on the GM.CD40L.CCL21 vaccine). At week 7, the patient underwent a biopsy of a liver lesion after cycle 1 day 1 of the vaccine. A moderate-to-high infiltration of CD3+ and CD45+ T cells is shown. *FNA* fine needle aspiration; *H&E* hematoxylin and eosin

We found no significant association between vaccine immunogenicity and outcomes. One patient treated with GM.CD40L.CCL21 underwent repeated biopsies as part of a work-up for pseudo-progression. Although this patient was ultimately determined to have progression by RECIST v1.1 and removed from treatment, immunohistochemistry staining from the posttreatment right kidney metastasis biopsy revealed a moderate-to-high number and density of CD45+ and CD3+ tumor-infiltrating lymphocytes (TILs) (Fig. 3).

Although only small numbers of patients had sufficient tissue for WT1 (21 patients), CEA (23 patients), hTERT (18 patients), and PD-L1 (16 patients) staining, we analyzed results for any potential associations with outcomes. No significant associations were observed between any of these proteins and PFS or OS (All *p* values > 0.05; Table 3).

**Table 3:** Tissue biomarkers and association with progression-free and overall survival

Biomarker	Number of samples	Progression-free survival				Overall survival			
		*p* value	Hazard ratio			*p* value	Hazard ratio		
			Point	95% CI			Point	95% CI	
				Lower	Upper			Lower	Upper
WT1 H-score									
0	13	Reference	Reference						
> 0	8	0.26	1.74	0.67	4.54	0.85	0.91	0.36	2.29
CEA H-score									
≤ 125 (median)	12	Reference	Reference						
>125 (median)	11	0.82	0.91	0.38	2.14	0.52	0.74	0.30	1.82
hTERT H-score									
≤ 100	12	Reference	Reference						
>100	6	0.96	0.97	0.35	2.69	0.65	1.28	0.44	3.74
PDL1 total proportion score									
< 50	9	Reference	Reference						
≥ 50	7	0.51	1.43	0.5	4.14	0.40	1.68	0.50	5.62

## Discussion

To our knowledge, this is the first trial to directly compare two vaccine strategies in patients with stage IV lung adenocarcinoma. We found that the GM.CD40L.CCL21 vaccine was well tolerated but did not clearly improve outcomes versus GM.CD40L vaccine alone. In addition, neither formulation seemed favorable, as median OS was 9.3 months with GM.CD40L versus 9.4 months with GM.CD40L.CCL21. Indeed, in this heavily pretreated, unselected patient population with lung adenocarcinoma, the median OS of GM.CD40L vaccine was comparable to results with chemotherapy and potentially comparable to some immune checkpoint inhibitors. In similar non-squamous NSCLC populations, nivolumab treatment showed median OS of 10.1 months (54.3% of patients with ≥ 3 prior lines) [4], and atezolizumab (425 patients) versus docetaxel (425 patients) treatment in the second-line (75%) or third-line (25%) setting showed median OS of 15.6 versus 11.2 months (*p* = .0015) [7]. In less heavily pretreated non-squamous NSCLC patients, median OS has ranged from 8.0 to 12.2 months for single-agent chemotherapy and from 9.4 to 15.6 months for anti-PD1/PD-L1 therapy [4, 5, 7, 11, 25]. Because our trial did not limit the number of prior lines of therapy, these cross-trials comparisons while intriguing pose limitations.

Although FDA-approved vaccines are not presently available for advanced/metastatic NSCLC treatment, the benefits of PD1/PD-L1 inhibitors demonstrated in NSCLC patients serve as proof of principle that harnessing the immune system can lead to an anti-tumor effect. Of note, only 20% of patients with NSCLC respond to single-agent PD1/PD-L1 inhibitors, suggesting intrinsic resistance mechanisms.

One strategy to improve “immunotherapy” could include combining the GM.CD40L vaccine (to expand the number of tumor-reactive T cells) with anti-PD1 therapy to allow T cells to remain functional when they enter into the tumor microenvironment. A multi-compartmental approach, at both the lymph-node level to enhance T cells and the tumor cell level, could overcome some resistance mechanisms and enhance outcomes. Treatment approaches that combine anti-PD1/PD-L1 therapies and the GM.CD40L vaccine may play a role in the advancement of combinatorial immunotherapy strategies. The low toxicity burden, in particular the lack of immune-related AEs with the GM.CD40L vaccine, could reduce the risk of overlapping toxicities when combined. Furthermore, the GM.CD40L vaccine uses a bystander cell approach, thus omitting the procedures necessary for DC generation ex vivo, including the need for apheresis, central line placement, and delays in administering the vaccine while cells are grown in culture. A trial combining the GM.CD40L vaccine and anti-PD1 is planned (NCT02466568).

Interestingly, a biopsy from one patient after treatment with GM.CD40L.CCL21 showed an abundance of TILs. CCL21 is known to induce chemotaxis of mature DCs and naïve T cells, and groups have demonstrated improved anti-tumor responses following intra-tumoral introduction of CCL21 through transduced DCs in mouse models [26, 27]. These findings may have been due to the addition of CCL21. The lack of significantly different clinical outcomes between the two treatment arms may be partly due to upregulation of both cytotoxic and regulatory T cells by CCL21, which in turn may have dampened responses. It remains reasonable to hypothesize that the GM.CD40L vaccine contributed to the abundance of TILs and, thus, remains a vaccine with potential effectiveness.

Our limitation of not having serial tumor biopsies for all patients prevented more extensive marker analyses. Although we observed no objective responses, our outcomes align with the previously published data mentioned above and represent a possible clinical benefit for patients with advanced NSCLC. The GM.CD40L vaccine cannot be used to personalize treatments based on an individual patient’s tumor antigens, and alternate vaccine approaches may be better suited to address these specific scientific inquiries. Still, because most patients are treated at non-academic sites, even in the setting of robust data, the feasibility of any given vaccine can certainly affect its broad uptake within the oncology community. The practicalities of the outpatient clinical setting should be kept in mind during product development.

## Conclusions

The addition of CCL21 to the GMCD40L vaccine was well tolerated but did not lead to improved effects. However, in limited biopsy analyses, one patient treated with GMCD40L.CCL21 displayed abundant TILs. This possible effectiveness warrants an additional study of the GM.CD40L vaccine in combination approaches.

### Electronic supplementary material

Below is the link to the electronic supplementary material.

Supplementary material 1 (PDF 233 KB)

## Abbreviations

AE: Adverse event
ATRA: All-trans-retinoic acid
CD40L: CD40 ligand
CI: Confidence interval
DC: Dendritic cell
DLT: Dose-limiting toxicity
ELISPOT: Enzyme-linked immunospot
FDA: US Food and Drug Administration
GM.CD40L.CCL21: GM.CD40L plus CCL21
GM-CSF: Granulocyte-macrophage colony-stimulating factor
H-score: Histology score
NCI: National Cancer Institute
NSCLC: Non-small cell lung cancer
OS: Overall survival
PFS: Progression-free survival
RECIST: Response evaluation criteria in solid tumors
RP2D: Recommended phase II dose
TIL: Tumor-infiltrating lymphocyte
TRAE: Treatment-related adverse event
