# Supplementary figures and images for: A phase I/randomized phase II study of GM.CD40L vaccine in combination with CCL21 in patients with advanced lung adenocarcinoma
Source: Cancer Immunol Immunother. 2018 Sep 12;67(12):1853–62. doi: 10.1007/s00262-018-2236-7 (PMC6244998; doi:10.1007/s00262-018-2236-7)

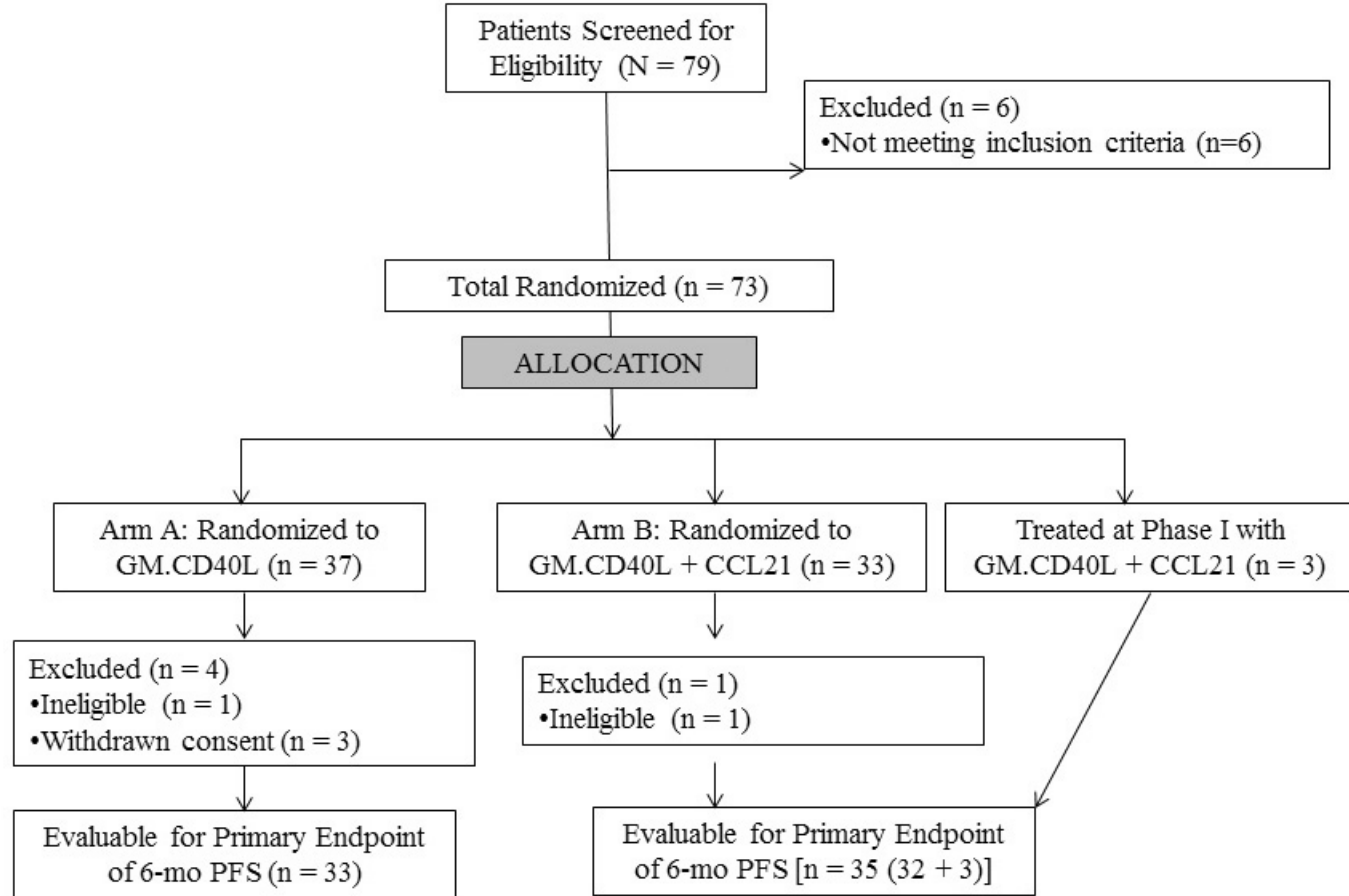

**Supplemental figure 1**

Supplement: Supplementary file 1 — Supplementary material 1 (PDF 233 KB) [file 262_2018_2236_MOESM1_ESM.pdf]
